# Advocating for the inclusion of kidney health outcomes in neonatal research: best practice recommendations by the Neonatal Kidney Collaborative

**DOI:** 10.1038/s41372-024-02030-1

**Published:** 2024-07-05

**Authors:** Kimberly J. Reidy, Ronnie Guillet, David T. Selewski, Marissa Defreitas, Sadie Stone, Michelle C. Starr, Matthew W. Harer, Namrata Todurkar, Kim T. Vuong, Semsa Gogcu, David Askenazi, Trent E. Tipple, Jennifer R. Charlton

**Affiliations:** 1 Division of Nephrology, Department of Pediatrics, Children’s Hospital at Montefiore/Albert Einstein College of Medicine, Bronx, NY 10467 USA; 2 Division of Neonatology, Golisano Children’s Hospital, University of Rochester, Rochester, NY USA; 3 Division of Nephrology, Department of Pediatrics, Medical University of South Carolina, Charleston, SC USA; 4 Division of Nephrology, Department of Pediatrics, University of Miami/Holtz Children’s Hospital, Miami, FL USA; 5 Department of Pharmacy, Children’s of Alabama, Birmingham, AL UK; 6 Division of Pediatric Nephrology, Division of Child Health Service Research, Department of Pediatrics, Indiana University School of Medicine, Indianapolis, IN USA; 7 Division of Neonatology, Department of Pediatrics, University of Wisconsin School of Medicine and Public Health, Madison, WI USA; 8 Division of Neonatal Perinatal Medicine, Department of Pediatrics, University of British Columbia, Vancouver, BC Canada; 9 Division of Pediatric Nephrology, Department of Pediatrics, Baylor College of Medicine, Houston, TX USA; 10 Section of Neonatal-Perinatal Medicine, Department of Pediatrics, Wake Forest University School of Medicine, Winston-Salem, NC USA; 11 Division of Nephrology, Department of Pediatrics, University of Alabama at Birmingham, Birmingham, AL UK; 12 Section of Neonatal-Perinatal Medicine, Department of Pediatrics, University of Oklahoma Health Sciences Center, Oklahoma City, OK USA; 13 Division of Nephrology, Department of Pediatrics, University of Virginia, Box 800386, Charlottesville, VA 22903 USA

**Keywords:** Outcomes research, Acute kidney injury

## Abstract

Acute kidney injury (AKI) occurs in nearly 30% of sick neonates. Chronic kidney disease (CKD) can be detected in certain populations of sick neonates as early as 2 years. AKI is often part of a multisystem syndrome that negatively impacts developing organs resulting in short- and long-term pulmonary, neurodevelopmental, and cardiovascular morbidities. It is critical to incorporate kidney-related data into neonatal clinical trials in a uniform manner to better understand how neonatal AKI or CKD could affect an outcome of interest. Here, we provide expert opinion recommendations and rationales to support the inclusion of short- and long-term neonatal kidney outcomes using a tiered approach based on study design: (1) observational studies (prospective or retrospective) limited to data available within a center’s standard practice, (2) observational studies involving prospective data collection where prespecified kidney outcomes are included in the design, (3) interventional studies with non-nephrotoxic agents, and (4) interventional studies with known nephrotoxic agents. We also provide recommendations for biospecimen collection to facilitate ancillary kidney specific research initiatives. This approach balances the costs of AKI and CKD ascertainment with knowledge gained. We advocate that kidney outcomes be included routinely in neonatal clinical study design. Consistent incorporation of kidney outcomes across studies will increase our knowledge of neonatal morbidity.

## Introduction

The advancement in our knowledge of neonatal kidney health including the epidemiology and impact of acute kidney injury (AKI) over the last decade is remarkable [1, 2]. AKI is a common morbidity in neonates and is often part of a multisystem syndrome that negatively impacts developing organs resulting in short- and long-term morbidities. Despite the high prevalence of AKI, data on kidney outcomes in neonatal studies remains infrequent and unstandardized. Furthermore, long-term kidney outcomes, such as chronic kidney disease (CKD), are infrequently ascertained in neonatal studies. To better understand how kidney dysfunction can impact other neonatal outcomes, it is critical to incorporate kidney-related data into study design.

**Fig. 1:**
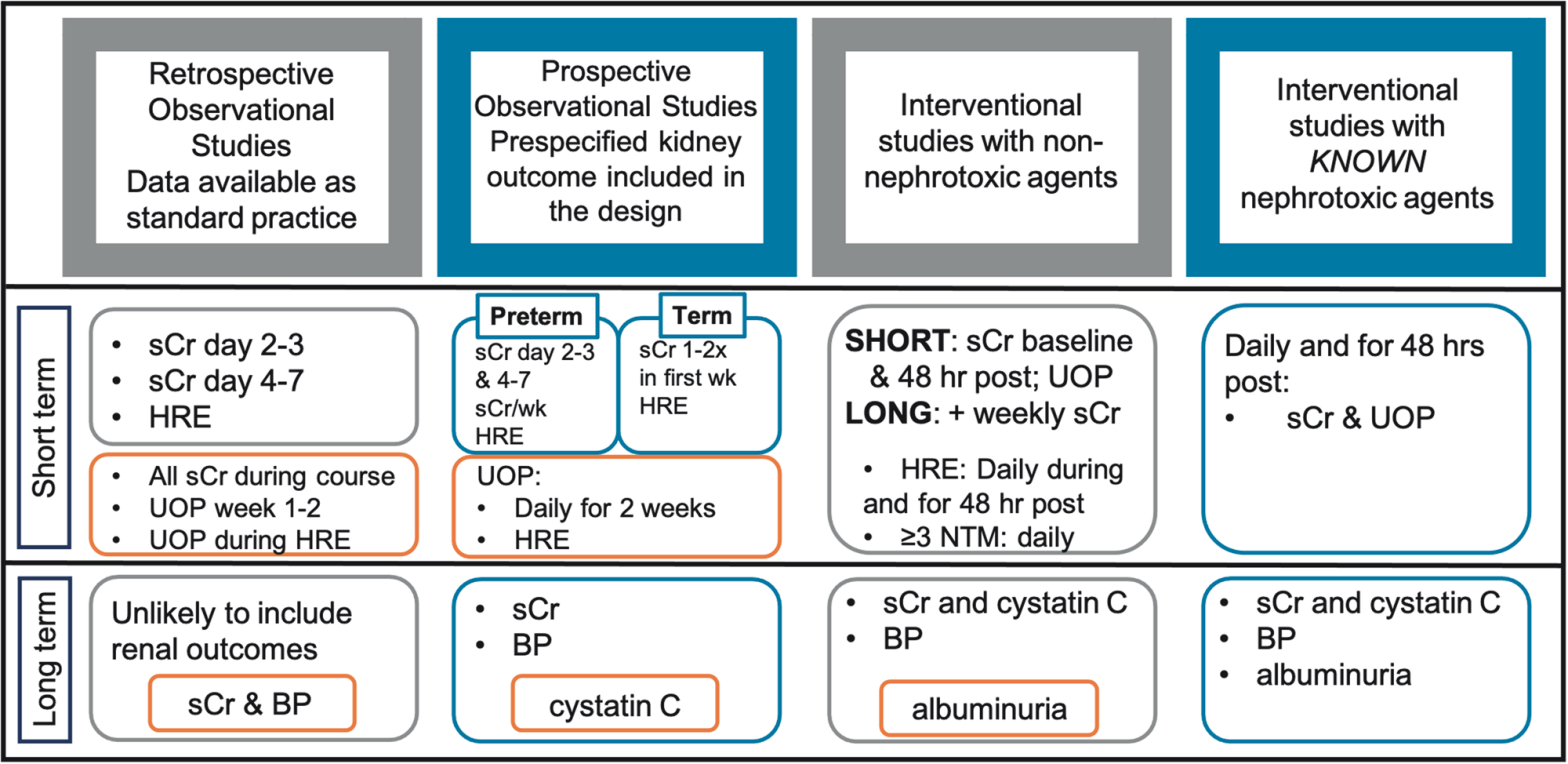
A tiered approach to incorporating short- and long-term kidney outcomes into neonatal studies. This approach attempts to balance the costs associated with ascertainment of AKI and CKD with the knowledge gained. We have stratified studies into four categories with the short-term outcome of acute kidney injury and the long-term outcome being chronic kidney disease or hypertension. We have outlined our recommendations in the same color as the study and highlight the ideal or optimal extra studies in orange boxes. High risk events include, but are not limited to, acquired neonatal intestinal disease (necrotizing enterocolitis (NEC) and spontaneous intestinal perforation), hemodynamically significant patent ductus arteriosus (PDA), sepsis, shock, surgery, and exposure to multiple nephrotoxic medications. Definitions: HRE high risk event, sCr serum creatinine, UOP urine output.

Below, we briefly review the current state of knowledge on short and long-term kidney outcomes in neonatal populations. We then propose best practice recommendations based on expert opinion for inclusion of short- and long-term neonatal kidney outcomes with supporting rationale for clinical studies in neonates. Inclusion of kidney outcomes will improve the primary study design and add to our knowledge of neonatal morbidity. We propose a tiered approach for the following types of neonatal studies: (1) observational studies (retrospective or prospective) limited to data available within a center’s standard practice, (2) observational studies involving prospective data collection where prespecified kidney outcomes are included in the design, and (3) interventional studies with non-nephrotoxic agents, and (4) interventional studies with known nephrotoxic agents (Fig. 1). We also provide recommendations for biospecimen collection when possible. These recommendations were collaboratively generated by a group comprised of neonatologists, pediatric nephrologists, clinical trialists, and pharmacists and represent consensus opinion based on current knowledge. These recommendations will need to be reassessed as data emerge on diagnostic approaches and new insights on acute and chronic kidney health in high-risk neonates is understood. This tiered approach is designed to be practical, balancing the costs of AKI and CKD ascertainment with knowledge gained.

## Review of the current state of evidence

### Short-term kidney outcomes

Over the past decade our understanding of the epidemiology, impact, and outcomes associated with neonatal AKI has significantly expanded [3]. Multiple single center studies have described the incidence and outcomes associated with neonatal AKI in a variety of high-risk populations, including preterm neonates and neonates with congenital heart disease, necrotizing enterocolitis (NEC), congenital diaphragmatic hernia, sepsis, or those requiring extracorporeal life support (ECLS) [4–13]. This work led to the international multicenter AWAKEN (Assessment of Worldwide Acute Kidney injury Epidemiology in Neonates) study, performed by the Neonatal Kidney Collaborative (NKC, www.babykidney.org) [3, 14]. AWAKEN included over 2000 neonates from 24 centers admitted over a 3-month period to neonatal intensive care units (NICU) [15, 16]. This retrospective study identified an overall incidence of neonatal AKI of 30% in high-risk patients across the spectrum of gestational age (GA) categories. Many risk factors for AKI were confirmed and several more were identified when this large study was stratified by GA and timing of AKI: mild-moderate kidney anomalies, urinary tract infections, and patent ductus arteriosus (PDA) [15, 16]. AKI was independently associated with increased morbidity (intraventricular hemorrhage [IVH], prolonged use of invasive respiratory support, length of stay) and mortality [3, 17–19]. The AWAKEN studies showed that AKI occurs more commonly than some other well recognized conditions associated with preterm birth including NEC and IVH.

Neonatal AKI negatively impacts pulmonary, neurodevelopmental, and cardiovascular health [3, 20]. As the short-term implications of neonatal AKI have become clearer, there has been an interest in evaluating the long-term impacts on other organ systems. This has been highlighted in secondary studies in AWAKEN that demonstrated an association between neonatal AKI, fluid balance, and pulmonary outcomes, including bronchopulmonary dysplasia (BPD) [18, 19, 21]. Starr et al. initially identified an association between AKI and a composite outcome of BPD or death in neonates born at 29–32 weeks’ GA (adjusted Odds Ratio (aOR) 4.21, 95% CI 2.07–8.61) [21] and then replicated this finding in a secondary analysis of the kidney outcomes from the Recombinant Erythropoietin for Protection of Infant Renal Disease (REPAIReD) study, an ancillary to the Preterm Erythropoietin Neuroprotection Trial (PENUT) [22]. This study of 885 premature infants born at 24–27 weeks’ GA showed that AKI, especially severe AKI, was associated with an increased odds of BPD grades 2 and 3 or death (aOR: 1.69, 95% CI: 1.16–2.46; aOR 2.05, 95% CI 1.19–3.54, respectively). More positive fluid balance during the first two postnatal weeks was also associated with poorer long-term pulmonary outcomes [22].

AKI has also been associated with adverse neurologic and neurodevelopmental outcomes in a variety of high-risk populations. In a single center study of 88 neonates treated with therapeutic hypothermia for hypoxic ischemic encephalopathy (HIE), Sarkar et al. identified an independent association of AKI with abnormal brain MRI findings obtained at postnatal 7-10 days (aOR = 2.9; 95% CI = 1.1–7.6) [4]. In a secondary analysis of the AWAKEN study of the 825 neonates <33 weeks GA, those with AKI were more likely to have had IVH (aOR = 1.6; 95% CI = 1.04–2.56). This was most prominent in infants born <28 weeks GA where AKI was associated with 1.9 times higher odds of having had IVH (aOR = 1.9; 95% CI = 1.08–3.23) [17].

### Long-term kidney and cardiovascular outcomes

Accumulating evidence suggests the long-term effects of prematurity on kidney health remains significant even after discharge from the NICU. In a recent study, 53% of extremely preterm neonates demonstrated at least one sign of CKD when examined at 2 years of age, with 18% having an estimated glomerular filtration rate (GFR) < 90 ml/min/1.73m^2^ [23]. In addition, 22% had systolic and 44% had diastolic blood pressure above the 90th percentile at the same 2 year follow up time point [23]. In large registry data sets and follow-up studies examining neonatal populations, individuals born preterm remain at increased risk for CKD, as well as adverse cardiovascular health outcomes, at all points across the lifecourse [24–27].

Beyond those born preterm, studies demonstrate other populations of NICU graduates also face an increased risk for CKD and poorer long-term cardiovascular health into adulthood. These include the individuals who have low birth weight, intrauterine growth restriction, congenital heart disease, or require ECLS [25, 28–33]. CKD is an independent risk factor for cardiovascular disease [34], and a reduced GFR may also modify outcomes of studies focused on the long-term health of other organs. Defining CKD in children is challenging, but important, as CKD is a potential confounder in studies examining the long-term health of other organs. Importantly, the risk for CKD is lifelong and data collection should be incorporated in studies beyond 2 years of age.

## Incorporating kidney outcome data in neonatal studies

To better understand the independent consequences of neonatal AKI on multisystem outcomes, it is critical to adopt a uniform definition of neonatal AKI and approach to the incorporation of kidney-related data into the design of neonatal clinical studies. Here, we provide the rationale and best practice recommendations for the integration of short- and long-term kidney outcomes into studies involving neonates. First, we will define kidney specific outcomes that we propose should be included in neonatal studies, including AKI and CKD. We then present a rationale and recommendation for short and long-term kidney health outcomes using a tiered approach as described earlier for the following types of neonatal studies: (1) observational studies (prospective or retrospective) limited to data available within a center’s standard practice, (2) observational studies involving prospective data collection where prespecified kidney outcomes are included in the design, (3) interventional studies with non-nephrotoxic agents, and (4) interventional studies with known nephrotoxic agents. We also provide recommendations for biospecimen collection when possible. These recommendations are based on current knowledge and represent a crucial first step in an iterative process.

## Defining kidney outcomes to be used in neonatal studies

### Neonatal acute kidney injury

**Recommendation**: We endorse the use of the neonatal modified KDIGO AKI definition **(**Table 1**)** to report neonatal AKI to facilitate collaboration and comparison across studies and populations. This definition includes both serum creatinine and urine output (UOP) criteria to diagnose and stage AKI.

**Table 1:** Short term (AKI) and long term (CKD) kidney outcome definitions.

AKI Stage	Serum creatinine	Urine output over 24 h	
**0**	No change in serum creatinine or rise <0.3 mg/dL	> 1 ml/kg/hour	
**1**	SCr rise ≥0.3 mg/dL within 48 h or SCr rise ≥1.5 to 1.9 x reference SCr* within 7 days	> 0.5 and ≤ 1 ml/kg/hour	
**2**	SCr rise ≥2 to 2.9 x reference SCr*	>0.3 and ≤ 0.5 ml/kg/hour	
**3**	SCr rise ≥3 x reference SCr * or SCr ≥2.5 mg/dL** or Receipt of dialysis	≤ 0.3 ml/kg/hour	
CKD Stage**	**GFR (ml/min/1.73 m2)**	**Persistent albuminuria**	
**1**	>90 with structural or functional abnormalities of the kidney or persistent albuminuria >30 mg/g	A1	<30 mg/g <3 mg/mmol
**2**	60–89	A2	30-300 mg/g 3-30 mg/mmol
**3**	3a: 45–59 3b: 30–44	A3	>300 mg/g >30 mg/mmol
**4**	15–39		
**5**	<15		

AKI is defined according to the neonatal modified Kidney Disease improving Global Outcomes (KDIGO) criteria.

CKD is defined based on the CKiD U25 formula.

*Reference SCr is the lowest prior SCr measurement; **In children and adults, CKD is defined by either a structural abnormality, persistent albuminuria >30 mg/g, OR GFR < 90 mL/min/1.73m^2^ for a period of 3 months and categorized into five stages. The presence of albuminuria (graded as A1-A3) increases risk for loss of kidney function.

**Rationale**: The optimal definition for neonatal AKI remains elusive and several definitions of AKI have emerged [35–37]. Changes in serum creatinine remain the current standard to diagnosis AKI, despite the well-known limitations. It is important to be aware of these limitations, particularly those that are unique to the neonatal population. Serum creatinine represents a marker of renal function, and kidney dysfunction is often detected 48–72 h after the injury has occurred. General limitations of serum creatinine to estimate GFR include that creatinine is not only filtered, but also undergoes tubular secretion. Creatinine is dependent upon muscle mass which is particularly problematic in neonates. There are several other unique limitations to using creatinine to estimate GFR in neonates including (1) fetal creatinine is influenced by placental clearance, (2) serum creatinine at birth reflects maternal values, and (3) measured GFR increases in the immediate postnatal period. GFR increases rapidly in the first two weeks following term birth due to increased cardiac output coupled with decreased renal vascular resistance leading to increased renal perfusion and filtration. In preterm infants, these challenges are exacerbated because GFR maturation likely differs by GA, and ‘normative’ data are lacking.

To overcome some of these challenges, Jetton et al. developed modified Kidney Disease Improving Global Outcome (KDIGO) criteria for neonatal AKI [38]. These consensus criteria were developed at the National Institutes of Health/National Institute of Diabetes and Digestive and Kidney Diseases (NIH/NIDDK) sponsored interdisciplinary workshop on Neonatal AKI in 2013 and have subsequently been utilized to define AKI in neonates in research and clinical care [39]. Although many other definitions for neonatal AKI exist [35–37, 40], the consistent use of the neonatal modified KDIGO AKI definition has fostered greater understanding of the incidence and impact of neonatal AKI.

UOP criteria are also included in the modified KDIGO criteria for neonatal AKI. We encourage the collection and use of UOP data to diagnose and stage AKI. Shortly after preterm birth in clinical practice, UOP is recorded in a rigorous manner. However, it is often difficult to obtain reliable urine quantification due to the presence of stool, and potential for leakage or evaporation. Invasive techniques such as catheters can cause harm and any adhesive devices, such as urine collection bags, are discouraged in immature neonates. UOP data usually are missing for a portion of the hospitalization and the accuracy of the UOP data have been questionable in some retrospective studies. Therefore, some retrospective studies have chosen to only use the serum creatinine criteria alone. It is important to recognize that eliminating the UOP criteria from the AKI definition will result in a lower detection rate of AKI [3]. When UOP is meticulously measured, the relationship between AKI and mortality increases substantially [3, 41]. In preterm infants, even relative oliguria with UOP < 1.5–2 mL/kg/hour is associated with increased mortality; future studies may identify new UOP thresholds that have even higher sensitivity for neonatal AKI diagnoses [41, 42].

### Long term kidney dysfunction

**Recommendation**: In adults and children, CKD is defined by a glomerular filtration rate (GFR) of <90 mL/min/1.73m^2^ for a period of three months and categorized into five stages (Table 1**)**. We recommend to assess estimated GFR at or after the age of 2 years using the U25 CKiD formula and including cystatin C in the calculation [43]. A calculator for the U25 formula can be found at https://ckid-gfrcalculator.shinyapps.io/eGFR/. The earliest signs of renal dysfunction can also include hypertension and proteinuria. We would recommend considering the inclusion of these variables as potential confounders in general neonatal follow up studies. We recommend defining hypertension as a persistent BP ≥95^th^%tile for age, sex and height [44]. We recommend that investigators consider an evaluation for albuminuria if the child is over the age of 2 years recognizing a paucity of normative data.

**Rationale**: To detect CKD in children, GFR estimation formulas were developed based on monitoring the rate of disappearance of exogenous or endogenous substances from the plasma, including the ‘bedside Schwartz’ and more recent U25 CKiD formula [43]. However, there are unique challenges in defining CKD in young children, particularly in children that were sick neonates. The populations used to develop the coefficients for GFR estimation were children with mild to moderate CKD over the age of 1 year [43]. The variables used to estimate GFR largely rely on height and serum creatinine. In children born preterm, linear growth can be compromised and serum creatinine can be falsely low due to decreased muscle mass. To overcome limitations of creatinine, non-muscle-based cystatin C, both independently and in combination with creatinine, is increasingly utilized to estimate GFR. Of note the cystatin C based eGFR formula does not include height in the formula. The most recent formula to estimate GFR in children (CKiD under 25, U25) does include a portion of children born preterm. Studies among preterm infants noted that measured GFR (mGFR) is best estimated when using a combined equation including both creatinine and cystatin C [45, 46]. Several studies have shown that formulas which incorporate cystatin C are more sensitive to detecting CKD, particularly in the preterm and low birth weight populations [47].

The optimal time for kidney function assessment remains unclear. However, healthy children attain rates equivalent to adult GFR (normalized to body surface area) by 2 years [48]. Thus, it is reasonable to assess GFR at 2 years of age by measurements of serum creatinine and cystatin C. As discussed above, in studies of neonates born <28 weeks of age, an estimated 18% exhibited decreased kidney function by 2 years of age [23].

#### Early signs of CKD

Proteinuria, albuminuria and/or hypertension can be early signs of CKD and incorporating these in neonatal studies have the potential to impact the interpretation of long-term health outcomes of neonatal studies [49, 50]. In the PENUT trial of neonates born <28 weeks gestation, 36% exhibited albuminuria by 2 years of age. Although a 24-hour urine collection for protein is the gold standard, this is not a feasible method in young children. Proteinuria is defined by urine protein/creatinine >0.2 mg/mg at or after the age of 2 years on a random urine collection. Albuminuria (defined as a urine albumin/creatinine ratio greater than 30 mg/g) is the gold standard for screening and staging CKD and is more specific to glomerular disease [51]. A first morning void is the ideal sample to assess for proteinuria or albuminuria because urine protein excretion can be affected by position [52]. It is also important to consider that proteinuria can be transient, ratios can be difficult to interpret if the urine is very dilute [52], and in those with very low muscle mass  there is a paucity of normative data. More data is needed to understand the role of albuminuria as a surrogate marker of long-term kidney health in neonates.

Hypertension is also an early sign of CKD [53] and has been shown to have an adverse effect on neurocognition [54]. There are several resources to measure the blood pressure correctly in children over 1 year old [55]. Ideally, blood pressure measurement requires proper positioning (seated with uncrossed feet flat on the ground, back supported and arm resting with the blood pressure cuff at heart level with the cuff on a bare right arm) [56] and at a minimum the patient needs to be calm. Correct cuff size is critical, and the proper cuff should be determined by measurement of arm circumference at the midpoint between the acromion and olecranon process [56]. The width of the cuff bladder should be at least 0.4 x the arm circumference [56]. The length of the bladder should be at least 0.8 x the arm circumference [56].

## Kidney data collection during NICU based on study type

To advance our understanding of the interaction between the developing kidney and other organ systems at various stages of development in the neonate, we propose a uniform approach to data collection in this population. Feasibility and cost must be balanced with the likely benefits to patient outcomes and research advancement. We propose a tiered approach to the collection of kidney specific data depending on the type of clinical research being undertaken (Fig. 1).

1. *Observational studies limited to data available within a center’s standard practice*
  **Recommendations**: At a minimum, we recommend focusing on extracting from the medical records the minimum and maximum serum creatinine at (1) two separate time points during the first postnatal week: days 2 or 3 and days 4–7 and (2) within 48 h surrounding high-risk events. High risk events include acquired neonatal intestinal disease (NEC and spontaneous intestinal perforation), hemodynamically significant patent ductus arteriosus (PDA), sepsis, shock, and surgery. If resources are available, the extraction of all clinically obtained creatinine concentrations and UOP would allow the best characterization of AKI as defined by the neonatal KDIGO definition. Uniform approaches and careful assessment of UOP are critical if UOP-based definitions are used.
  **Rationale:** In observational studies, where data are limited to the scope of a center’s standard practice, AKI most often occurs within the first two weeks after birth and in association with high-risk events [15, 16]. We recognize there is no current consensus on what constitutes standard of care monitoring for AKI. However, during the development of a U01 grant application, 15 academic centers agreed that serum creatinine measurements should be standard of care during the first week and during high-risk events (personal communication). Furthermore, weekly creatinine levels allow for the determination of a baseline to serve as a comparison during an episode of clinical instability (e.g., sepsis, NEC). In addition, we endorse extracting the minimum and maximum serum creatinine surrounding clinically significant events (within 48 h) that are known to be associated with AKI, including NEC, hemodynamically significant PDA, sepsis, shock, surgery (eg. cardiac repair requiring bypass or major abdominal surgery) and in the setting of multiple nephrotoxic medications. Without serial measurement of creatinine, episodes of AKI may be missed as AKI is often clinically silent and may not be accompanied by a decrease in UOP [15, 16]. Investigators can use these data to diagnose AKI using the neonatal modified KDIGO criteria (as recommended above) in addition to noting if there was a clinical diagnosis of AKI by the care team.
  It is important to note that AKI is often under-recognized by clinicians [12] and relying on ICD-10 coding of AKI episodes may limit detection [57, 58]. Other important kidney-related data to consider collecting include the presence of hypertension (persistent blood pressure readings ≥95^th^ percentile for postmenstrual age and/or treatment with antihypertensive medications) [59, 60] and urinary tract infections. Long-term kidney outcomes are often not included in this type of study design, however, can be considered based on hypotheses and specific aims if outpatient medical records are available.
  Disorders of fluid balance often occur in neonates, including those with critical illness as well as those with AKI [61]. While not currently included as part of the KDIGO neonatal AKI definition, disordered fluid status often indicates an inability of the kidneys to maintain homeostatic balance [62]. To more completely characterize kidney function, we recommend obtaining weight measurements (ideally daily during the first 1–2 weeks and then frequently thereafter) and calculating fluid balance using an anchor weight or birthweight (during the first 14 post-natal days) [62]. Fluid balance can be calculated using the following equation:
  $$\frac{[{{{{{\rm{Current\; weight}}}}}}\left({{{{{\rm{kg}}}}}}\right){-}{{{{{\rm{Anchor\; Weight}}}}}}\left({{{{{\rm{kg}}}}}}\right)]\, \times \, 100 \% }{{{{{{\rm{Anchor\; Weight}}}}}}({{{{{\rm{kg}}}}}})}$$
2. *Prospective observational studies that include kidney outcomes in the design*
  **Recommendation for assessment for short term kidney outcomes (AKI)**:
  When short term kidney outcomes are included in the design of the study, the GA of the neonate should be considered. For preterm neonates, we recommend that serum creatinine be measured (1) serially in the first two weeks after birth with a minimum of once during days 2 or 3 and once during days 4–7, (2) weekly, and (3) during high-risk events. For term neonates, we recommend 1–2 times in the first week and within 48 h of high-risk events. If creatinine is increasing, we suggest collecting values daily until resolution (return to baseline or a nadir). Optimally, AKI is best defined by collecting all available serum creatinine levels. We recommend collecting daily UOP (ml/kg/hr) data for the first two weeks and for 48 h following high-risk events. If UOP is decreased ( < 1 mL/kg/hr), continue to monitor until resolution (UOP ≥ 1 mL/kg/hr).
  **Rationale:** The recommendations proposed here have been incorporated into the National Institute of Child Health and Disease (NICHD) Neonatal Research Network (NRN) Generic Database (GDB). Beginning in January 2024, all neonates born at 27 weeks’ GA or less, born in an NRN center, have clinically obtained kidney-related data recorded. It was estimated that the cost in coordinator time for this additional data collection would average $15/GDB-eligible neonate, depending on the complexity of the clinical course (*personal communication)*. A sample data form of kidney data included in the GDB can be found in **Appendix**. A budget justification for the collection of this data can be found in Table 2. Inclusion of these data in the NRN was felt to be justified as it allows for a more accurate estimate of the prevalence of neonatal AKI, the association of AKI with significant episodes of clinical instability, and association with 2-year neurodevelopmental outcomes. Equally important, AKI may be a confounder for other study outcomes, such as mortality, length of stay, and BPD [3, 17, 18].

**Table 2:** Budget justification for the collection of kidney outcomes. The data extraction was tested by four independent study coordinators and three independent study coordinators.

Category of complexity	Number based on 2020 data (%)	Minutes to extract data by category	Total time per year (h)
“uncomplicated”	373 (20%)	15	93
“usual complexity”	974 (53%)	20-30	324 - 487
“complex course”	508 (27%)	45-60	381-508
All GDB	1855 (100%)	20-30	620-930
Cost (@$40/hr)			$25-37,000/year

Uncomplicated NICU course

Neonates discharged prior to 36 weeks

“Usual” degree of complexity

Neonates discharged after 36 weeks but prior to 120 days

Extended stay, complicated course

Neonates in the NICU for more than 120 days
  Beyond preterm neonates, monitoring serum creatinine and UOP is also recommended for other neonatal populations requiring intensive care who are at high risk for AKI, such as infants hospitalized for HIE, congenital heart disease, surgical evaluation or those receiving multiple nephrotoxic medications [3]. Researchers should be aware that close monitoring of serum creatinine concentrations has led to improved detection and successful prevention of neonatal AKI in initiatives such as the Baby NINJA project. In the Baby NINJA quality improvement study, neonates exposed to a high burden of nephrotoxins were identified [63] and monitoring of creatinine was suggested, but not mandated, in the nephrotoxin-exposed neonates. This attention to kidney health led to a 78% reduction of AKI and in the sustainability era of the study nephrotoxin medication exposure decreased [63].
  **Recommendation for assessment of long-term kidney outcomes (CKD):**
  We recommend that an assessment of kidney function and blood pressure monitoring be incorporated into all follow-up studies involving NICU graduates, coordinating with study protocol visits and timelines. The presence of CKD should be assessed at the age of 2 years or older using the U25 formula, including creatinine and cystatin C. Of note, obesity is a known additional risk factor for the development of proteinuria, CKD, and hypertension in preterm born children [64, 65]. Hence monitoring growth trends, body mass index and obesity diagnoses may also be considered in longitudinal studies.
  **Rationale:** The lack of longitudinal studies limits conclusive recommendations, but substantial epidemiologic evidence supports increased risk of hypertension and CKD in children born preterm [25, 30] and in populations of neonates diagnosed with AKI [32]. As discussed above, hypertension and CKD are risk factors for other adverse outcomes, including poor neurocognitive function [66–69] and cardiovascular disease [55, 70].
  The optimal time for kidney function assessment remains unclear, however, healthy children attain rates equivalent to adult GFR (normalized to body surface area) by age 2. As discussed above, in studies of neonates born <28 weeks’ GA, up to 18% exhibited decreased kidney function by 2 years of age [23]. Thus, it is reasonable to assess GFR at 2 years of age by measurements of serum creatinine and cystatin C.
3. *Interventional studies with non-nephrotoxic agents*
  **Recommendation:** In interventional studies, data collection about kidney health should be pre-planned and used in the final analyses to assess impact of AKI on study outcomes. It is important to consider: (1) AKI events may affect the bioavailability of the drug and (2) AKI may have an independent effect on the primary or secondary outcomes of the study. Importantly, this data collection will also estimate the effect of the study intervention on kidney health. We recommend the same data collection for short term kidney outcomes as described for prospective observational studies, with intensive screening for AKI during first 2 weeks of life and following high-risk events with ongoing screening but less intensive screening following the high-risk event in all GA groups. Data collection may be modified based on the timing and pharmacokinetics/pharmacodynamics of the planned intervention, the study hypothesis, and specific aims. If the investigational drug is short-acting (half-life <24 h) and/or is given for a limited duration ( < 72 h), then a baseline and single post-intervention assessment of serum creatinine within 24–48 h of the intervention along with UOP assessment for 48 h should be sufficient. Long-acting or more chronic ( > 72 h) interventions require a tailored approach to monitor for AKI. A reasonable approach would include monitoring by serum creatinine and UOP for the first week following initiation, followed by a minimum of weekly serum creatinine assessment until 1 week after the intervention is complete. As with prospective observational clinical studies, serum creatinine (and UOP, if feasible) should be monitored for 48 h following high risk events. In addition, monitoring of serum creatinine after nephrotoxic drug exposure (high number or long exposure) should be included in study design. In studies that have a follow up component, we encourage the inclusion of long-term kidney health assessment at 2 years of age. This would include measurement of eGFR (by U25 formula [43] with creatinine and cystatin C) and BP at a minimum, but optimally would also include a urine albumin/creatinine ratio.
4. *Interventional studies involving agents/devices suspected or known to be nephrotoxic*
  **Recommendation:** For an investigational drug that may be nephrotoxic, a baseline serum creatinine is recommended prior to initiation of the intervention. Frequent (optimally, daily) measurement of serum creatinine is recommended throughout the intervention period and for a minimum of 48 h after study drug is discontinued. If the investigational drug is long acting (half-life>24 h) and/or is given for a prolonged duration ( > 72 h), we recommend daily serum creatinine and UOP monitoring for a minimum of 1 week, followed by weekly serum creatinine to enable the accurate diagnosis of AKI. In addition, if a subject concomitantly receives ≥3 nephrotoxic medications during the study period (Table 3) a daily serum creatinine should be obtained for at least two days after exposure to the study drug. Similarly, if the study subject has a high-risk event as described above during the study period, daily monitoring of creatinine levels should be considered for 48 h. If AKI occurs, daily serum creatinine should be obtained until 48 h post AKI resolution (return to baseline serum creatinine). In addition, a diagnosis of AKI should lead to repeat assessment of kidney function (at a minimum by measurement of serum creatinine) 1–3 months after the event [71]. Depending upon the duration of NICU stay, this may be via a serum creatinine at time of hospital discharge. In addition to these measures, we support biobanking of blood and urine if feasible to facilitate future ancillary studies of known and new experimental biomarkers in relation to the study drug [72–75]. Approaches to biobanking are discussed in more detail below. We also strongly recommend the inclusion of long-term kidney health assessment following nephrotoxic interventions by assessing eGFR (U25 formula with creatinine and cystatin C), BP, and albuminuria, ideally at 2 years of age.

**Table 3:** Likely concomitant nephrotoxic medications in neonatal study subjects.

Medication	Half- life	Mechanism of action	Mechanism of renal injury	Mechanism of elimination
Acyclovir	• Short (term neonates: 3 h)	• Substrate and specific inhibitor of herpesvirus DNA polymerase	• Crystal nephropathy due to poor solubility in urine which may lead to tubular obstruction	• Renal: Primarily by GFR with a small contribution from tubular secretion
Amphotericin B deoxycholate, amphotericin B liposomal	• Short (infants/children: 18.1 h)	• Fungicidal via binding to ergosterol of the lipid bilayer of the fungi and disrupting membrane permeability, leading to loss of anions and glucose	• Acute tubular necrosis: increased tubule permeability, electrolyte wasting, and glomerular damage/filtration impairment secondary to drug binding to cholesterol in cell membranes	• < 5% excretion via renal and biliary routes; continues to be renally eliminated as it is released from tissues and is detectable in urine for 7+ weeks after last use
Aspirin	• Short (adult: 3 h)	• Irreversible inhibitor of COX-1 and 2 enzymes which leads to decreased formation of prostaglandin precursors and inhibited platelet aggregation	• Interstitial nephritis	• Hydrolyzed by esterases in GI tract, blood, and synovial fluid to salicylate which is then metabolized via hepatic conjugation; excreted in urine
Captopril, enalapril	• Short (infants with CHF captopril: 1.2–12.4 h; enalapril: 10.3 h)	• Competitive inhibitor of angiotensin-converting enzyme which leads to increased plasma renin activity and reduced aldosterone secretion	• Hemodynamic AKI: increased efferent blood flow	• Hepatic metabolism to metabolites (captopril) or active drug (enalapril → enalaprilat), then excreted in the urine as unchanged drug and metabolites
Ganciclovir, valganciclovir	Short (neonates ganciclovir: 2.4 h; infants valganciclovir: 3.5 h)	Competitively inhibits to binding of deoxyguanosine triphosphate to DNA polymerase which leads to inhibition of viral DNA synthesis	• Crystal nephropathy: drug precipitates in renal tubules	• Valganciclovir converted to ganciclovir by intestinal mucosal cells and hepatocytes; ganciclovir excreted in the urine as unchanged drug
Gentamicin, tobramycin	• Short (neonates gentamicin: 3–11.5 h; neonates tobramycin: 2–9 h)	• Inhibit bacterial protein synthesis by binding to the 30 S ribosomal subunit	• Acute tubular necrosis: accumulation within proximal tubule cells is directly cytotoxic • Reduction of GFR via tubuloglomerular feedback mechanism	• Renal
Ibuprofen, Indomethacin, ketorolac	• Short (6 mo-2 yrs ibuprofen: 1.8 h; neonates indomethacin: 11–20 h; >6 months ketorolac: 4 h)	• Reversible inhibitor of COX-1 and 2 enzymes which leads to decreased formation of prostaglandin precursors	• Hemodynamic AKI: reduced afferent blood flow	• Hepatic metabolism; primarily excreted in urine, a small amount in the feces
Iodine-containing contrast (i.e. iohexol, ioversol)	• Short but may be prolonged	• Opacifies vessels and anatomic structures in the path of flow of the contrast media	• Acute tubular necrosis	• Renal: unchanged in the urine
Nafcillin	• Short (about 2 h for neonates/infants)	• Beta-lactam antibiotic which inhibits bacterial wall synthesis	• Acute interstitial nephritis: (allergy-mediated, not dose-dependent)	• Hepatic metabolism; primarily excreted in feces, about 30% via urine as unchanged drug
Piperacillin/ tazo-bactam	• Short (3.5 h)	• Piperacillin: beta-lactam antibiotic which inhibits bacterial wall synthesis • Tazobactam: inhibits beta-lactamases	• Acute interstitial nephritis (allergy-mediated, not dose-dependent) • Acute proximal tubular necrosis	• Renal: primarily as unchanged drug
Vancomycin	• Short (term neonates: 6.7 h)	• Glycopeptide antibiotic which inhibits cell wall synthesis of gram-positive bacteria	• Acute tubular necrosis (caused by oxidative stress on proximal tubular cells or, less commonly, proximal tubule obstruction from formation of casts)	• Renal: unchanged in the urine via GFR ( ≥ 90%)

**Rationale for interventional studies:** Neonatal nephrotoxic medication stewardship and AKI surveillance are paramount considerations for the design of nephrotoxic or potentially nephrotoxic interventions to characterize the effect on kidney health. Further, as discussed above, AKI events may influence the study outcomes. We recommend monitoring for AKI in interventional studies involving agents not known to be nephrotoxic, as well as known nephrotoxic agents. This will allow identification of previously unrecognized adverse events and more appropriate attribution of study outcomes to the intervention alone.

Surveillance of renal function can be tailored based on the known properties of the drug under investigation. Although neonatal pharmacologic development has improved in recent decades; there continues to be a relative paucity of drug trials in neonates when compared to the older pediatric population [76, 77]. Hence, off-label utilization of drugs with limited pharmacokinetic (PK) and pharmacodynamic (PD) data is extremely common in the NICU [78, 79]. The ongoing paucity of neonatal drug trials is multifactorial and involves issues related to research ethics, limited funding opportunities, strict regulations, and the uniquely dynamic physiologic adaptations in neonates that impact drug PK and PD [80].

The monitoring of creatinine and timely diagnosis of AKI will support real-time clinical adjustment of concomitant medications to the investigational drug that require renal dosing. The renal excretion of drugs depends on the sum of glomerular filtration, tubular secretion, and reabsorption. As well as GFR maturation discussed above, tubular functions undergo rapid postnatal adaptations and maturational changes in the neonate and infant. The rate of drug metabolism is usually lower in neonates and maturational pharmacokinetic changes occur with advancing GA and weight. Moreover, using a weight-based approach for drug dosage calculations can be complicated by the initial weight loss experienced by neonates and the subsequent period of weight gain [81]. Ongoing maturation of organs that metabolize drugs such as the liver occurs over the neonatal period and must be taken into consideration. As discussed above, accurate assessment of GFR is challenging and this creates significant barriers for the determination of physiological endpoints related to neonatal renal drug elimination capacity [82]. This is especially true in preterm infants where maturation of renal function is delayed. Despite the overall lack of evidence-based data for dosing, Neofax® and Lexicomp® are highly utilized resources that incorporate adjustment of renal dosing based on GA and body weight.

**Examples of neonatal studies that have incorporated kidney outcomes:**

Several large neonatal studies have or have plans to incorporate kidney related outcomes. These include PENUT/REPAIRed, the ongoing NICHD supported NRN PDA study, and the NIH-funded NICU Antibiotics and Outcomes study (NANO, NCT03997266). These interventional studies involve both nephrotoxic and non-nephrotoxic agents. The PENUT/REPAIReD study, an NIH/NIDDK-funded ancillary collection of kidney data in the context of a randomized clinical trial of erythropoietin for neuroprotection in preterm neonates, allowed protocolized documentation of kidney and other organ-specific short and long-term outcomes [23, 83].

The ongoing NRN study of optimal management of PDA will help investigators explore the potential association between different PDA management strategies and kidney health and the contribution of AKI to longer term outcomes. Incorporation of kidney-related data is particularly important as both the physiology inherent in a PDA as well as some of its potential therapies may have significant impacts on the developing kidney. Although the analysis of retrospective data from AWAKEN suggested that PDA management approach does not affect the incidence or severity of AKI, there were many inherent limitations to that study [84]. Incorporating data collection into the randomized clinical trial of expectant vs. active management of PDA will address many of these limitations.

The NANO trial aims to compare the rate of adverse outcomes of empiric exposure to antibiotics vs placebo at birth in neonates less than 31 weeks’ GA [85]. To be eligible for inclusion in the study, neonates cannot be at high risk for early onset sepsis (where antibiotics would clearly be indicated), nor delivered for purely maternal reasons and at essentially no risk (where antibiotics would be very unlikely to be prescribed). In addition to the composite incidence of NEC, late onset sepsis and death, the collection of kidney-related data will enable investigators to assess the influence of the study intervention on AKI as well as the contribution of AKI on the other adverse outcomes. In particular, extraction of clinically obtained creatinine levels around episodes of late onset sepsis and NEC will allow incorporation of presence or absence of AKI in multivariate analyses of the study’s primary outcome.

## Development of kidney outcome biorepository

**Recommendation**: Whenever feasible, we recommend that biospecimen collection be incorporated into study protocols and design. Blood, urine, and saliva can be collected for novel biomarker analysis or stored in a biobank in anticipation of future identification of more sensitive and specific markers of kidney function and exploration of effects of gene variants on outcomes.

**Rationale**: Developing biorepositories that include blood, urine, and saliva is critical to provide opportunities for clinical advancement. These biologic samples can be stored for future ancillary kidney specific outcomes in neonatal research. This investment is already being supported in many small and large scale funded research projects nationally and internationally (PENUT [83]/UK Biobank [86]). These sample collections are minimally invasive and can expand our understanding of proteomic, metabolomic and genomic variation in kidney outcomes for at-risk neonates. Recently, urinary neutrophil gelatinase-associated lipocalin (NGAL) was approved by the US Food and Drug Administration for use in AKI detection in infants and children aged three months and older admitted to the ICU. While urine NGAL values are affected by preterm birth and are not yet approved for those <3 months, some studies have demonstrated the ability of urine NGAL to predict AKI in certain neonatal populations [87]. Non-invasive methods of urine collection include use of a cotton ball in the diaper or an adhesive urine bag, with new devices on the horizon. Researchers should be aware the collection methodology and processing methods may have an impact on the analysis of the urine in neonates [88]. This may influence how urine is collected and stored in a biorepository. We anticipate that active research in the field will likely identify new blood and urine biomarkers in the future, and thus biobanking has the potential to add lasting value to both observational and interventional studies for future exploratory ancillary neonatal kidney research.

## Conclusions

Assessment of both short- and long-term kidney outcomes should be incorporated into neonatal research studies. AKI is a common short-term kidney complication experienced by neonates requiring intensive care that modifies the risk for dysfunction in other developing or immature organs. Broad implementation of AKI assessment in clinical studies of neonates using readily available clinically obtained data will not only improve the primary study design but will add globally to our knowledge of neonatal morbidity. Neonatal studies that include a long-term follow-up should incorporate metrics of kidney outcomes, including eGFR, BP, and albuminuria assessment as hypertension and CKD can confound other long term study outcomes. In addition, leveraging the use of biorepositories in prospective observational and interventional studies for future measures of novel biomarkers in the urine and blood would be advantageous. We advocate for this tiered approach depending upon study design and objectives to balance the costs of kidney outcomes ascertainment with the knowledge gained.

## Appendix

**SAMPLE DATA FORM**

**Table:**

1. Clinical diagnosis of AKI	Y/N
a. If yes, date of diagnosis	MM/DD/YYYY
b. Inpatient consultation or outpatient referral to nephrology?	Y/N
2. Blood pressure at status	___mmHg
3. Diagnosis of hypertension	Y/N
a. If yes, were antihypertensives used?	Y/N

Maximum and minimum clinically-obtained creatinine levels weekly for the first 6 weeks:

**Table:**

	Maximum creatinine		Minimum creatinine	
	Date	Concentration	Date	Concentration
Week 1				
Days 1-4				
Days 5-7				
Week 2				
Week 3				
Week 4				
Week 5				
Week 6				

**Table:**

1. If late onset sepsis, was creatinine measured during the first 10 days after diagnosis?	Y/N
a. If yes,	Highest value_____________mg/dL MMDDYYYY
2. Was the infant diagnosed with Urinary tract infection:	Y/N
a. If YES,	Organism _________________ MMDDYYYY
4. Weight at each of the time points not already recorded (3, 7, 14, 28 days)	Y/N
a. If YES,	Highest value____________mg/dL MMDDYYYY
3. If proven NEC, was creatinine measured in the first 10 days after diagnosis?	
Day 3	_________ gm
Day 7	_________ gm
Day 14	_________ gm
Day 28	_________ gm

**GDB MOP additions:**

Clinically obtained creatinine levels:

 Enter the highest and lowest creatinine concentrations over each of the indicated time periods. If only one creatinine was measured, enter that value and date in both columns.

 If no creatinine was measured during the time period, indicate “not done” (from drop down menu).

NG03 E. INFECTION

 If the baby was diagnosed with late onset sepsis, indicate if at least one creatinine was measured within 10 days of the diagnosis.

 If measured during that time, enter the highest level obtained and the date it was measured

 Indicate if the baby was diagnosed and treated for a urinary tract infection (UTI).

 If yes, enter the date and the organism (see Appendix for code)

NG03 F. Gastrointestinal

 If the baby was diagnosed and treated for proven NEC (Bell’s stage 2 or 3), indicate if at least one creatinine was measured within 10 days of the diagnosis.

 If measured during that time, enter the highest level obtained and the date it was measured.

NG07 Respiratory

 On days 3, 7, 14, and 28, in addition to the respiratory support being provided, record the weight in grams on that day.

NG03 Kidney **(NEW SECTION)**

 Enter “YES” if the clinical team diagnosed the baby with acute kidney injury (AKI) at any time during the NICU stay.

 If AKI was diagnosed, indicate if the clinical team either requested an inpatient pediatric nephrology consultation or made a referral for an outpatient evaluation by pediatric nephrology.

 Enter the blood pressure (systolic and diastolic) measured within ± one week of “status”.

 Enter “YES” if the baby was treated for hypertension with any of the following: amlodipine, captopril, enalapril, enalaprilat, hydralazine, isradipine, labetalol, lisinopril, nicardipine, propranolol
